# The Frontotemporal Degeneration (FTD) Clinical Research Learning Institute

**DOI:** 10.1093/geroni/igaf122.019

**Published:** 2025-12-31

**Authors:** Shana Dodge, Meaghan Sharp, Wanda Smith, Lauren Massimo

**Affiliations:** Association for Frontotemporal Degeneration, King of Prussia, Pennsylvania, United States; University of Pennsylvania, Philadelphia, Pennsylvania, United States; CalHHS Alzheimer’s Disease & Related Disorders Advisory Committee, Sacramento, California, United States; University of Pennsylvania, Philadelphia, Pennsylvania, United States

## Abstract

Recent years have seen a marked increase in research opportunities for frontotemporal degeneration (FTD), a common cause of early-onset dementia that impacts behavior, communication, and/or motor functioning. Recruitment and retention for trials, however, remains a challenge, as do community misconceptions about FTD research. The Parkinson’s and ALS fields have seen success tackling these barriers through patient engagement initiatives designed to empower people directly impacted by neurodegenerative conditions with critical information about clinical research. Utilizing a curriculum adapted from the Northeast ALS Consortium (NEALS), in 2024, the Association for Frontotemporal Degeneration (AFTD) and University of Pennsylvania Frontotemporal Degeneration Center (Penn FTD Center) piloted an FTD Clinical Research Learning Institute (CRLI). The one-day, virtual FTD CRLI received 70 applications for 12 training spots, demonstrating a community interest in research training. The FTD CRLI program included sessions on FTD drug development and clinical trials, ethics and informed consent, study design and statistics, how to read research papers, personal experiences with research, and FTD advocacy. Graduate “research ambassadors” are given opportunities to participate in initiatives that influence and improve the FTD research process and are encouraged to bring awareness of FTD research to their own communities. On a post-training survey, ambassadors reported an eagerness to connect research information to the general public and increased enthusiasm for practical advocacy guidance, AFTD and the Penn FTD Center are expanding FTD CRLI opportunities for 2025 with a focus on diversifying the pool of research ambassadors.

